# Is it Genocide? Gaza, Ukraine, and Other Crimes Against Humanity

**DOI:** 10.1007/s11673-024-10411-6

**Published:** 2025-02-28

**Authors:** Paul James

**Affiliations:** Institute for Culture and Society, Western Sydney University, Locked Bag 1797, Penrith, NSW 2751 Australia

**Keywords:** Genocide, Violence, Crimes against humanity, Extermination, Israel-Palestine, Gaza

## Abstract

In the context of the current war, the question “Is the Israeli state effecting genocide in Gaza?” suggests a threshold legal excursus, a definitional contestation, or a cry of moral outrage. This article does not take any of those paths. It lives the pain of the unethical deaths of tens of thousands of civilians in Gaza, while beginning the longer-term task of seeking a way beyond deploying the concept of “genocide” as a performative gesture of shock and horror. The article argues that the meaning of genocide is being emptied out by an unsettling of the grounding conditions of political debate and the relativization of political language. While the evidence is strong that crimes against humanity are being perpetrated in Gaza, both by the Israeli state in its attack upon civilians and by Hamas in holding hostages, the provisional ruling by the International Court of Justice that there is a case to be answered is the most resolute that we can be at this point. Clearly, the war has to stop. In the meantime, the article suggests an alternative way of naming the horror.

My mind revolts, unresolved on some questions, clearer on others. Perhaps it is a necessary kind of irresolution, one that derives from the nature of the situation. In the context of what is happening in Gaza, one question, at least, requires the kind of intellectual distance that at this time is almost impossible. Is it Genocide? At one level, the question seems irrelevant as the news comes in daily of more tragedies, more deaths, with once lively neighbourhoods reduced to rubble, tank-tracks, and mangled bodies. And yet the question is everywhere, too often turned into a statement of fact. Genocide is in the courts, the petitions, and the voices of people demonstrating around the world. This means that the contours of any irresolution need to be clear. And, as someone deeply critical of the current situation, I need to be explicit about what needs to be done. Ethical complexity cannot be answered by deferral or quiescence.

## Posing the Question

The question of what is happening in Israel/Palestine can be posed very directly. Is the Israeli state effecting genocide in Gaza? Without the wisdom of Solomon, I will not attempt to arrive at an elegantly simple answer or a threshold test. In any case, the question depends upon the evidence in relation to the meaning of genocide, emphasizing the systematic and abiding intention to destroy an ethnic community for itself. It cannot be judged by the ethical urgency of the horrors in Gaza; nor can it depend upon numbers in a necro-accounting of those confirmed slain. Rather, it is conditional upon naming the perpetrator precisely, determining what they did (are doing), and discerning what they intend. This means distinguishing genocide from ethnic cleansing, from domicide, and from extermination. These are all crimes against humanity, but only genocide seeks deliberately to destroy an ethnic group *for itself*.

The mass violence in Gaza is simply and unaccountably horrific. And there is no doubt that the violence is directed against civilians, intentionally or unintentionally—Israeli and Palestinian, but predominantly Palestinian, as it has always been since 1948. Those words—intentionally or unintentionally—hang heavily. Whatever the case, the war has been and is being fought in a brutally unethical way, first on that terrible day of October 7, and then on every subsequent terrible day—days and months of brutal violence. As the title of the essay foreshadows, I am convinced from the evidence that continuing crimes against humanity are being perpetrated in Gaza by both the Israeli government/military and by Hamas—just as crimes against humanity are being perpetrated in Ukraine and Sudan. However, for all of this, at the time of writing the charge of genocide in Gaza remains, at least for me, still to be proven. In this, I agree with the careful sentiments of the Jewish-American scholar, Omer Bartov, written early in the war:

As a historian of genocide, I believe that there is no proof that genocide is currently taking place in Gaza, although it is very likely that war crimes, and even crimes against humanity, are happening. That means two important things: First, we need to define what it is that we are seeing, and second, we have the chance to stop the situation before it gets worse. We know from history that it is crucial to warn of the potential for genocide before it occurs, rather than belatedly condemn it after it has taken place. (Bartov 2023)

What would count as proof that is more than a pastiche of quotes?

Even when a secular politician such as Benjamin Netanyahu instrumentalizes an ancient creed that talks of total retaliatory destruction, it remains circumstantial. His words were these: “you must remember what Amalek has done to you, says our Holy Bible. And we do remember” (Netanyahu 2023). In mythical stories, the Amalek were the first people to attack the Jewish people after the Exodus. According to the Bible, God promised to completely wipe out the memory of Amalek from the earth, and to wage an eternal war with Amalek across every generation. This is *herem*; it reads as a brutal command to genocide. Indeed, but across the centuries, Jewish scholars have grappled with this passage—some avoiding its horror, and some suggesting that it is allegorical, pointing to a final act that will occur outside of time at the end of history (Feldman 2004). Whatever be the case, it is clear that Netanyahu is being an all-too-clever rhetorician, whistling variously to different current sensibilities. Every year in the Jewish calendar on the sabbath before *Purim* the passages called *Parashat Zachor* referring to the Amalek are read. For some Jews, it is a time of confronting their own evils from the past and present, repenting on injustice, and seeking reconciliation. For others it is an ongoing call to arms in a world that will always seek the destruction of the Jewish people. And for a few, both sensibilities hold at the same time.

Proof would entail more than quoting the Israeli Minister of Defence, Yoav Gallant, while leaving out the first four sentences of the following statement:

You saw [on 7 October] what we are fighting against. We are fighting against human animals. This is ISIS of Gaza. This is what we are fighting against. Gaza won’t return to what it was before. We will eliminate everything. If it doesn’t take one day, it will take a week. It will take weeks or even months, we will reach all places. (Gallant 2023)

It is relevant that he was later sacked as Minister of Defence allegedly because he could not defend continuing the war against Hamas as it had been fought.

## Emptying Out the Meaning of Genocide

Despite the care and restraint in some voices, the concept of “genocide” is too often instrumentalized with sad abandon. It is used to shock. It is used because the violence perpetrated by the Israeli military is so shocking. It is used because people are wanting to invoke “the worst of all crimes.” It is used thinking that a concept will galvanize people to action. It is used thinking instrumentally that this horror provides a time for recruiting new believers to an old cause—with the ends justifying or vindicating the rhetorical means. And it is used all too loosely in articles by groping intellectuals in genocide journals because the place of publication assumes the subject of their documentation and argument. There must be an ethics of language-use in war—an extension of *jus in bello*—and it should apply to all. Thus, for all that this war requires deep and utter condemnation, the every-which-way whoosh of rhetorical missiles sent into the contemporary debates have produced a post-ethical Manichean controversy out of which there can *at the moment* be no *positive* reconciliation.

On the one hand, the term “genocide” is being emptied out of its defined meaning, much like the way in which the concepts of “fascist” or “terrorist” have become ill-defined and contested terms of abuse. The deployment of such terms has now too often become a performative gesture, a marker for identifying oneself with a particular politics or understanding of a situation. “Genocide,” “fascist,” and “terrorist” are not *essentially* contested concepts like “democracy” or “work of art”: that is, appraisive, internally complex, contestably non-contradictory, and open (Gallie 1955). Oh, how anachronistic that notion of “essentially contested concepts” has become in a world where the epistemological grounding of truth has become increasingly relativized. “Genocide,” like “fascist,” gives the impression of still being a grounded-but-contested term. But it has been lifted out of the sphere of contested rounds of evidence-based dialogue into the relativized world of thin, shock-based contestation. It is not that such terms cannot be deployed. However, at least in the argument that I am presenting here, they need to be re-embedded in grounded defendable narratives.^1^

On the other hand, the concept of genocide is still part of the ambiguous regime of juridical judgement where definitions still matter. As such, the International Court of Justice could do little else but respond to South Africa’s 2023 submission with studied irresolution: “at least some of the acts and omissions alleged by South Africa to have been committed by Israel in Gaza appear to be capable of falling within the provisions of the Convention.” Legally distant words. Careful words. And so, to the judgement: “In light of the foregoing, the Court concludes that, *prima facie*, it has jurisdiction pursuant to Article IX of the Genocide Convention to entertain the case and that, consequently, it cannot accede to Israel’s request that the case be removed from the General List” (International Court of Justice 2024). This judgement means that the case will go now to adjudication by the Court. They are backwards words, but not twisting and turning like the dissembling rhetoric currently used by governments and militaries to defend war crimes that they are committing.

This, I suggest, is the kind of deferred adjudication that we have necessarily come down to. It is decried by Zionists who do not recognize the authority of the court. And it is ignored by many in the Pro-Palestinian movement as they use the same words as “evidence” to say that it must be genocide. This is a world in which the semi-Solomonic wisdom of the International Court of Justice is lost in rhetorical leafleting no better that the propaganda dropped into Gaza from Israeli planes. As I write, the latest leaflet shows a photograph of the dead Hamas chief Yahya Sinwar. If this is a contemporary perversion of *memento mori* where its classical meaning “remember, you must die” has taken an ugly turn, it is a heartless one sent to the living, most of whom disavow Sinwar’s politics.

In this area, however, juridical meaning is also being emptied out as the unintended consequence of political intent. For example, in 1992, the United Nations General Assembly deemed *en passant* that ethnic cleansing is a form of genocide:

Gravely concerned about the deterioration of the situation in the Republic of Bosnia and Herzegovina owing to intensified aggressive acts by the Serbian and Montenegrin forces to acquire more territories by force, characterized by a consistent pattern of gross and systematic violations of human rights, a burgeoning refugee population resulting from mass expulsions of defenceless civilians from their homes and the existence in Serbian and Montenegrin controlled areas of concentration camps and detention centres, in pursuit of the abhorrent policy of “ethnic cleansing”, which is a form of genocide. (United Nations 1992)

Here the term “genocide” was thrown into a preamble concern that had no tested legal standing. And there has been confusion ever since. The process has thus been doubly confused by such institutions deeming some acts genocidal even if they are not part of an *inextricably* connected larger pattern. It is impossible in such a short essay to take this apart, but it is used here as an exemplar of the mess. A few years later the evidence came out that the Bosnian detention camps, as horrific as they were in 1992, were nothing like the Nazi concentration camps of the 1940s. And the ethnic cleansing, as horrific as it was, was massively overstated by the world’s press. More to the point, the worst crimes tragically took off after the massive NATO bombing of 1999, but by then the guilt of the world had been largely sated. The bombing, causing more civilian deaths, allowed the NATO politicians to say, “we have done something.”

## Unsettling the Ground Beneath the Naming of Genocide?

In summary, it *is possible* that genocide is occurring in Gaza, and this should be questioned, investigated, and responded to through a precisely enunciated precautionary principle. This *positive* irresolution suggests that South Africa’s taking of the Israeli government to the International Court of Justice with the charge of genocide is ethically defensible. The point that the Israeli government allowed or possibly instrumentalized either genocidally oriented language or language that might incite genocide in the context of mass killing gives the case a *prima facie* force, even if it is not sufficient evidence in itself for judgement.

Such an argument should not, however, be taken to mean that one has to agree that genocide is currently taking place in Gaza—even if we are seeing what some call “domicide,” a version of infrastructure-based ethnic cleansing, the wiping out of people’s homes either to force them to move on or to achieve a political end. It is even possible that if the world was not watching the situation might descend into genocide. The same has been alleged in relation to the Russian invasion of Ukraine (though the Ukrainian case is tenuous). But that just adds another level of complexity. In the current world of genocidal legislation and aversion, when would a government publicly signal its intention to such an act?

Perhaps, given all of this, in the immediacy of an existential crisis, the confidential South African Memorial of October 28, 2024 is all we can hope for.^2^ That, and more importantly a firm call for a ceasefire, the cessation of all arms trading with Israel, and the beginning of a productive and tough dialogue about the future of Palestine and Israel. Here we are still on solid ground—the ground of defined meaning. Unfortunately, on the other hand, the ground beneath the current dominant use of the concept of “genocide” is deeply unsettled, even when it is used to call forth anguish because nothing else seems to convey the horror with sufficient force. However well intentioned, this naming, has the effect of relativizing meaning in a way that parallels the dominant trend towards a post-truth world of right-wing populism. In this unsettled world, just as big little lies turn into post-truth conjuration, signified horror turns can turn into hollowing incantation.

Along the way to thinking through this mess, I find myself confronted by a cognate series of complicated and controversial questions. Should the term “holocaust” be preserved for the Jewish Holocaust? What are the consequences of treating the Holocaust as singular? Is what is happening in Gaza a parallel to what happened in Nazi Germany? The last question is made all the more tragic by the issue that the Jewish people, once the victims of unspeakable violence, are now being used as rhetorical “human shields” by a right-wing Jewish state arguing for the “necessity” of “inadvertently” massacring civilians in the task of annihilating a political enemy. It is politicide.^3^

Even asking these questions has been unsettled by a Manichean politics. But we should be able to ask them. It must be possible to negotiate a world of grounded meaning in which one can say more than one thing at the same time. Take the following statements: the Jewish holocaust was genocide; the extermination of the Tutsis in Rwanda was genocide; the war crimes in Bosnia were not genocide, even if there is circumstantial evidence that Radovan Karadžić may have had genocidal intent; the war crimes in Gaza, while having some limited parallels to the crimes against humanity in Nazi Germany, are not genocide. And neither are the crimes in Ukraine, however horrific they are in directly targeting civilians. Unless we are now dealing with a league table of horror, it should be possible that those statements are equally true, and that we can condemn each of them for themselves, and with deep practical consequences.

## Beyond Defending the Indefensible

Writing in his heartfelt foreword to a book with the title *Is the Holocaust Unique?* the Jewish Zionist, Israel Charny, expressed what appears to be a perfect rendition of the problem:

I object to any statement that in any way minimizes the significance or sacredness of any people’s losses, even when the understandable and legitimate purpose is to honor one’s own people’s tragedy, or even the specific tragedy of any other given people that one is respectfully researching or is committed to remembering. I also object to scholars who transform the material of genocide scholarship predominantly into definitional arguments about what is or is not “genocide.” I call this “definitionalism,” or such an intense concern with establishing the boundaries of a definition that the reality of the phenomenon—in this case, human tragedy and infamy—is banished to a secondary position and no longer genuinely experienced. (Charny 2009, xi).

The fracturing of Charny’s position emerges, however, as he elaborates his stance on Gaza. He quickly enters the dangerous territory of self-confirming contradiction. In a recent article, Charny proposes a new legal conception of self-defence that, in effect, seeks to legitimize war crimes: “Israel is fighting back legitimately in Self-Defense in Response and in Self-Defense against Future Genocidal Attacks that Employ Citizens as Human Shields. The Geneva Conventions specifically outlaw use of human shields and justify fighting back in response. Self-Defense does not include genocidal intent” (Charny 2024). Yes, using human shields is a war-crime, but there is no modern ethical framework in which one crime legitimates another (of even worse proportions), especially not a temporally projected crime that *might possibly happen.* Here we also enter the territory of a *traditional* cosmological ethics of an “eye for an eye” suffused with *modern* illogic. That is, it argues that we can legitimately take your eye out to make it easier for us defend ourselves from a future possible attack by you.

Another problem is that, for all Charny’s concern about definitionalism, we still need a working definition of genocide. Like the definitions of the singular embodied crimes of rape, murder, and so on, different crimes against humanity need to be defined in order to act against them. The obvious answer is that a Jewish scholar, Raphael Lemkin, writing immediately after the Jewish Holocaust gave us such a working definition. Unless we choose to live in a world of relativized meaning, it works as well as such definitions can work:

… “genocide” means any of the following acts committed with intent to destroy, in whole or in part, a national, ethnical, racial or religious group, as such: (a) Killing members of the group; (b) Causing serious bodily or mental harm to members of the group; (c) Deliberately inflicting on the group conditions of life calculated to bring about its physical destruction in whole or in part; (d) Imposing measures intended to prevent births within the group; (e) Forcibly transferring children of the group to another group. (International Criminal Court 2021).

The shaky issues here turn on the difficulty of putting boundaries around the phrases “in part” “intent to destroy” and “as such.” And these all become questions of qualitative judgement that need to be determined case by case.

One of the pathways to answering the question of genocide in Gaza that I considered was to go back to Johan Galtung’s writing on structural violence—*structural*, meaning the outcome of patterned practices where intention is irrelevant to the judgement. Writing a couple of decades ago, Galtung suggested that “The prognosis today is the same as fifty years ago: oscillation between the structural violence of occupation, exploitation and alienation and the direct violence of bombs and bombing, terror and torture” (2000, 131). Using this parallel process for thinking about genocide seemed to me to answer my questions for a while. Does what we have witnessed in Israel/Palestine over the past four-score-and-seven years amount to a *structure of genocide*? What we have seen is certainly a structural condition of fast, then incremental, then inexorable, and now horrific displacement of Palestinians based on their non-Jewishness. It might well be called “ethnic cleansing,” a purposeful policy designed to remove by violent and other means the civilian population of another ethnic or religious group from certain geographic areas. However, for all that fast and slow horror, the sticking point in calling it genocide is that what the perpetrators clearly intended was displacement by many means, rather than systematic removal of this *ethnie* from the face of the earth.

Sometimes the displacement was through killing civilians, but other times it was by processes that appear as banal as the passing piece of planning legislation, the reworking of a definition such as “defence,” or the planting of a copse of trees. For example, Saree Makdisi documents how the language of sustainability has been used by the Israeli government to plant trees over the ruins of once-Palestinian villages, thus hiding unspoken crimes and expunging a history of ethnic cleansing. This present-absence of the Palestinian people in Israel is profound. It is produced by an instrumental abstraction of the process of *habeas corpus*, literally meaning “you shall have a body,” that has underpinned modern democracy and law for a couple of centuries. In an apparently banal example of structural violence, David Kishik, an Israeli-born philosopher, takes us to an Israel/Palestine after 1948 where Palestinians will have sometimes be deemed to have consequential bodies and other times will have those bodies abstracted away from them by an act of law:

The Palestinians who fled their villages during the war but returned after the battles subsided were rather surprised to discover that the Israeli army had taken full control of their property. The authorities told them that even though they were indeed “present” within the boundaries of the Israeli territories, as far as their lands, homes, shops, bank accounts, stocks, artworks, and so forth were at stake, they were considered “absentees.” Though these “internal refugees” were physically present in the courtrooms where the repeatedly appealed to reclaim what was previously theirs, in the eyes of the law they were, at the very same time, absent. In official legal documents they were therefore referred to—with what must be one of the most colossal disregards of basic common sense in the history of jurisprudence—as “present-absentees.” (2012, 78)

However, as much as this abstracts from the embodied realties of people’s lives, even with the qualifier “cultural” or “legal” this is not genocide.

## Establishing the Meaning of Basic Terms

Crimes against humanity, according to the Rome Statute of the International Criminal Court, include the following acts: murder; extermination; enslavement; deportation or forcible transfer of population; imprisonment or other severe deprivation of physical liberty in violation of fundamental rules of international law; torture; rape; sexual slavery; enforced prostitution; forced pregnancy; enforced sterilization, or any other form of sexual violence of comparable gravity; persecution against any identifiable group or collectivity on political, racial, national, ethnic, cultural, religious, gender; enforced disappearance of persons; apartheid; and other inhumane acts of a similar character intentionally causing great suffering, or serious injury to body or to mental or physical health—with all of these themselves requiring careful definition. All of these are legally specified acts that come under the single term, “unethical.” What is needed is for the international community to sort out the complexity of all these terms into relational definitions that hold in concert with each other (see Table 1 below for an initial attempt).

**Table 1:** Forms of “crimes against humanity”

Primary category	Other categories	Definition	Associated Statute	Qualifier
Crimes Against Humanity Crimes against humanity are defined as unethical acts of embodied assault, committed knowingly by the perpetrator as part of a widespread or systematic attack directed against any population or ecological region. This includes ethnic cleansing, extermination, slavery, torture, and ecocide, and domocide.	Violent Crimes Against Humanity	Violent crimes against humanity are unethical acts of embodied violence, committed knowingly by the perpetrator as part of a widespread or systematic attack directed against any population.	• Nuremberg Charter, 1945. • Rome statute of the International Criminal Court, 1998.	Here, the definition has broadened from crimes *in* war to all crimes where systematic unethical violence is knowingly perpetrated. I would delete the adjective, “civilian” from the 1998 Rome statute and add the words “or region” thus include acts of democide that are otherwise left out of the larger category.
	War Crimes	These are crimes against humanity committed in war-time.	• The Geneva Conventions	
	Genocide	This process comprises acts committed with an intent to destroy, in whole or in part, a national, ethnical, racial, or religious group as such.	• Genocide Convention, 1948.	This still requires an “intent to destroy” a group “as such,” in whole or in part.
	Extermination	Extermination is an event or process involving a mass killing of persons where the act is treated instrumentally as the means to another end. Its Latin origin, *exterminare*, means “to drive beyond the boundaries”	• Rome statute of the International Criminal Court, 1998.	Though not accidental or without the foreknowledge of the perpetrator, it does not require proof of a specific intent to destroy the group “as such” in the way that genocide does. In this definition however, different from the Rome Statute, killing is a requirement for the concept to pertain.
	Ethnic cleansing	Ethnic cleansing is a purposeful policy and practice designed to remove by violent and other means the civilian population of another ethnic or religious group from certain geographic areas.	• Security Council Resolution 780	This term has no agreed definition under international law.
	Domicide	This is the widespread and intentional destruction of people’s homes and basic infrastructure.	No associated statute or formal recognition.	This term has no agreed definition under international law.

This table goes against some of the current dominant conventions of international law, and in some cases extends beyond them. Most significantly, it questions why crimes against humanity are often distinguished from war crimes, with both crimes bundled under the category of *mass atrocity crimes* when a war crime can be singular and not mass. The mess here is in part because the category of war crimes has a long ethical history, and others do not. This cacophony of terms has accumulated historically as an unresolved accumulation of international law that does not have a single home. There is a pressing need to sort this all out, defining the terms in relation to each other but also giving agreed legal definition to terms like “ethnic cleansing” and “domicide” which continue to float in and out of contention without legal standing.

My immediate sense in relation to Gaza is that many commentators want to broaden the definition of genocide to make it co-extensive with politicide, domicide, and other crimes against humanity. Why? Why is it so important that this word “genocide” covers everything? Unlike the definition of refugee which was originally so narrowly defined because of political reasons that it required decades of legal amendment, the definition of genocide was broad to begin with (from *gens* or ethnos, abstracted from kin-related). And it does not preclude recognizing other forms of horror and mass violence that are equally bad.

At the same time, the Israeli and Russian government can justifiably be accused of crimes against humanity and state terrorism against civilians, the same domicidal tactics that Winston Churchill used in the German fire-bombings of World War II and Harry Truman used in the atomic bombing of the citizens of Hiroshima in 1945, except that this process has extended across years and years, taking many forms, many of which never reach the global news.

## Conclusion

All of these means that we need to be careful to use the terms carefully, ethically, and not empty them out by political misuse. It does not require the accusation of genocide to end the war. There are many alternative measures, including immediately ceasing all military aid to Israel. In the transition to another world, Netanyahu and certain members of the Israeli government and military should be tried for crimes against humanity. And we should seek to move on to a fragile peace of reconstruction, juridical resolution, and ongoing reconciliation, recognizing the legitimacy of both countervailing claims to a home in Israel/Palestine. As Ben Okri writes:

History, in its wisdom, in its terror,

Has brought us to this place

To this impossible mathematical

Equation where we cannot solve

The future with the past,

With blood or blame or bombs

Or unsustainable slogans.

I see a new future is possible there.

I see the lands fertile in tough

Invaluable collaboration. (2024)

Obviously, Ben Okri’s poem is as unresolved as the present essay. But some things are clear. He is against both unsustainable slogans and bombs of submission. He is critical of black-letter juridical or abstract mathematical arguments about what constitutes the horror. And he still hopes for a future where human collaboration will get us out of this tragedy.

For all of this, in the transition to another world or at least until the formal adjudication of the International Court of Justice, we still need a name for what is happening in Gaza. What then should we call this process of treating the Gazans as abstract others, as nonconsequential means to another end, as expendable beings in a war on terror? It can be called *extermination*. Perhaps this is worse than genocide. In human history, extermination is truly a crime of the recent present. It systematically abstracts and instrumentalizes other humans as no more than things in the way. It says that “they” have no consequence. By comparison, at least the Ameleks are mythologized, feared, and ritualized as a people of consequence. The same could not be said for the Palestinians. They have been rendered as abstract others without human standing. They have come to be treated as *nothing* people.

## Data Availability

Not applicable

## Footnotes

1 See for example, James 2020, where the author argues among other things that Donald Trump is a fascist.

2 https://dirco.gov.za/south-africa-delivers-evidence-of-israel-genocide-to-icj/

3 The charge of the war in Gaza being one of *politicide* is one which the Israeli government would proudly concede. But it does raise another question: How has the world arrived at such a point where it is possible to use the politicidal threats of another to legitimize one’s own war crimes?
